# Elimination of refractory ventricular tachycardia storm and fibrillation using stereotactic radiotherapy

**DOI:** 10.1002/ccr3.6690

**Published:** 2023-01-16

**Authors:** Alexander Wutzler, Borris Tiedke, Mohamed Osman, Noha Mahrous, Reinhard Wurm

**Affiliations:** ^1^ Department of Cardiology Klinikum Frankfurt (Oder) Frankfurt (Oder) Germany; ^2^ Cardiovascular Center St. Josef Hospital, University Hospital of the Ruhr‐University Bochum Frankfurt (Oder) Germany; ^3^ Department of Radiation Oncology Klinikum Frankfurt (Oder) Frankfurt (Oder) Germany

**Keywords:** radioablation, stereotactic ablation, ventricular storm, ventricular tachycardia

## Abstract

Ventricular tachycardia storm is a potentially lethal condition with limited treatment options. Failed ablation is associated with a fourfold mortality increase in this population. Stereotactic body radiotherapy was proposed in these cases. We report a case where radiotherapy was safely performed, leading to the elimination of adequate shocks.

## Graphical abstract

Radiotherapy offers a feasible, safe, and effective therapy to VT storms refractory to pharmacotherapy and ablation. Radiotherapy can be therapeutic, palliative, or a bridge to transplant. More studies are needed to further investigate the use of SBRT.

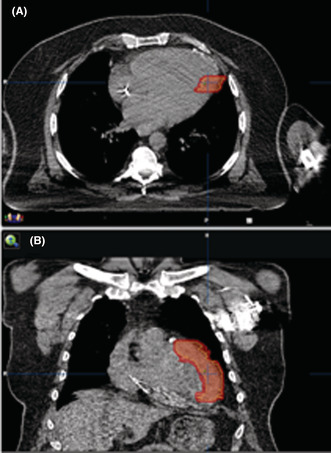

## 1 INTRODUCTION

Ventricular tachycardia (VT) storms and ventricular fibrillation (VF) are arrhythmias with limited and unsatisfactory treatment options and are associated with high mortality. Failed catheter ablation poses a fourfold increased risk of death in patients with VT/VF.^1^ Furthermore, heart failure and adequate implantable cardioverter‐defibrillator (ICD) shocks are associated with impaired prognosis.^1^ Stereotactic body radiotherapy (SBRT) has been proposed as a last resort in patients with VT storm and VF refractory to medical therapy and catheter ablation.^2^, ^3^

## 2 CASE REPORT

**FIGURE 1:**
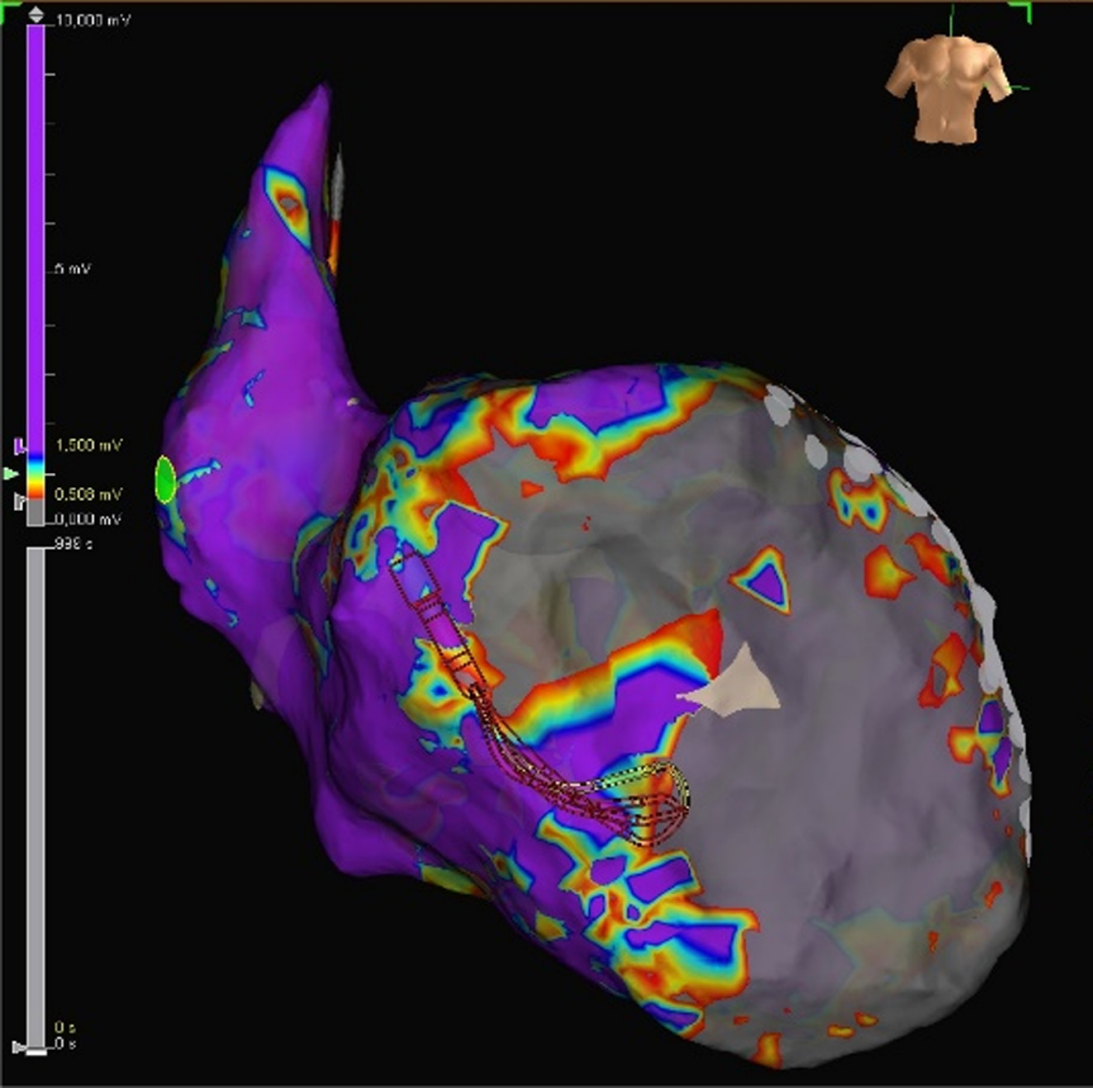
Electroanatomical map of the left ventricle in Anteroposterior view

**FIGURE 2:**
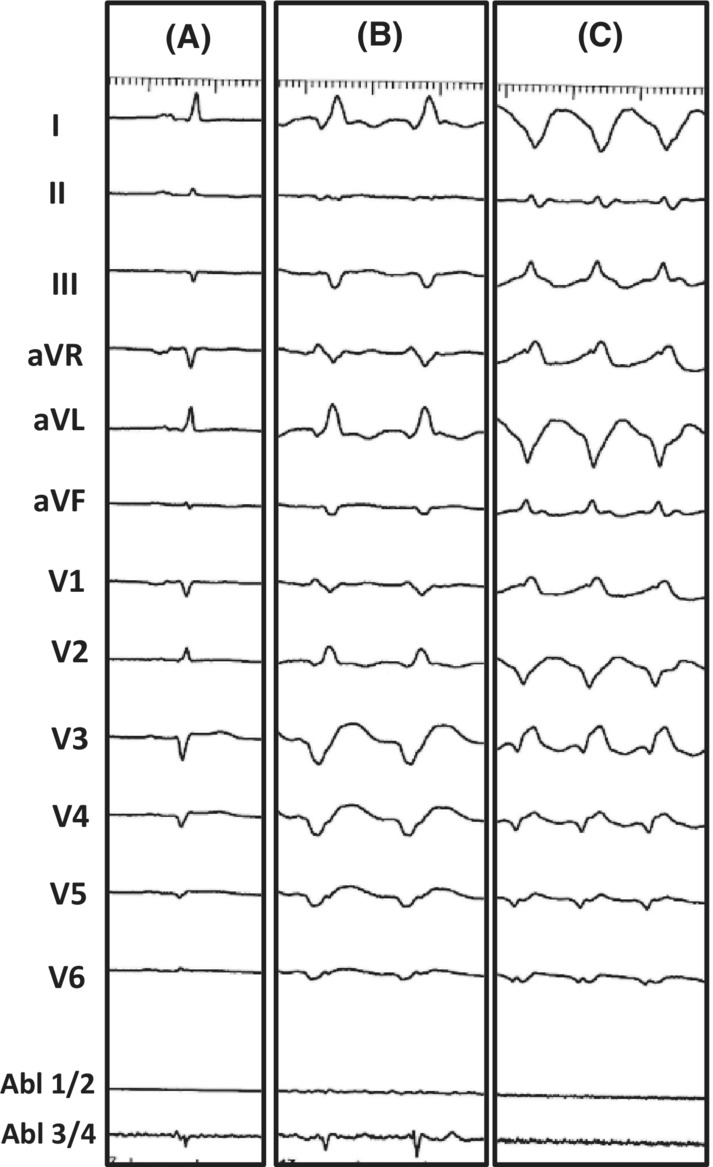
Panel A: 12‐ Lead baseline ECG. Panel B: ventricular tachycardia morphology number 1. Panel C: ventricular tachycardia morphology number 2

A 56‐year‐old male patient with a history of myocardial infarction and heart failure presented to our hospital with cardiac arrest due to a VT storm that degenerated to ventricular fibrillation. The VT storm was refractory to pharmacotherapy. The patient experienced a previous episode of VF 10 years ago, following which he underwent multiple revascularizations of the left anterior descending (LAD) coronary artery. He had a left ventricular aneurysm ever since with decreased left ventricular ejection fraction (LVEF) (15%). He underwent ICD implantation 7 years ago. ICD Interrogation revealed multiple VT and VF episodes within the last 6 weeks, which were treated with ATP or adequate ICD shocks. After resuscitation and stabilization, the patient underwent coronary angiography and the patient had a proximal LAD lesion as well as in‐stent restenosis of a preexisting LAD stent. We implanted two drug‐eluting stents and drug‐eluting balloon dilatation was performed. Antiarrhythmic therapy with Lidocaine and Amiodarone was begun. Unfortunately, the hemodynamically unstable ventricular tachycardias persisted and we decided to perform catheter ablation of the VT. Electroanatomical mapping revealed anterior, septal, and apical extensive scar areas in the left ventricle with multiple areas of late potentials [Figure 1]. Catheter ablation was performed, and substrate modification as well as ablation of two ventricular tachycardia morphologies, was performed [Figure 2]. The first VT was ablated at the inferior apical area of the left ventricle and was rendered non‐inducible. We induced a second morphology that was ablated at the mid‐ anterior wall. This tachycardia degenerated into a different morphology during ablation. The latter was terminated with burst stimulation. Finally, the VTs were non‐inducible. Our goal was to decrease the VT burden as well as the number of adequate shocks. Nonetheless, the VT recurred after 3 months. Due to therapy—refractoriness, extensive substrate, and involvement of the septum, SBRT was performed.

**FIGURE 3:**
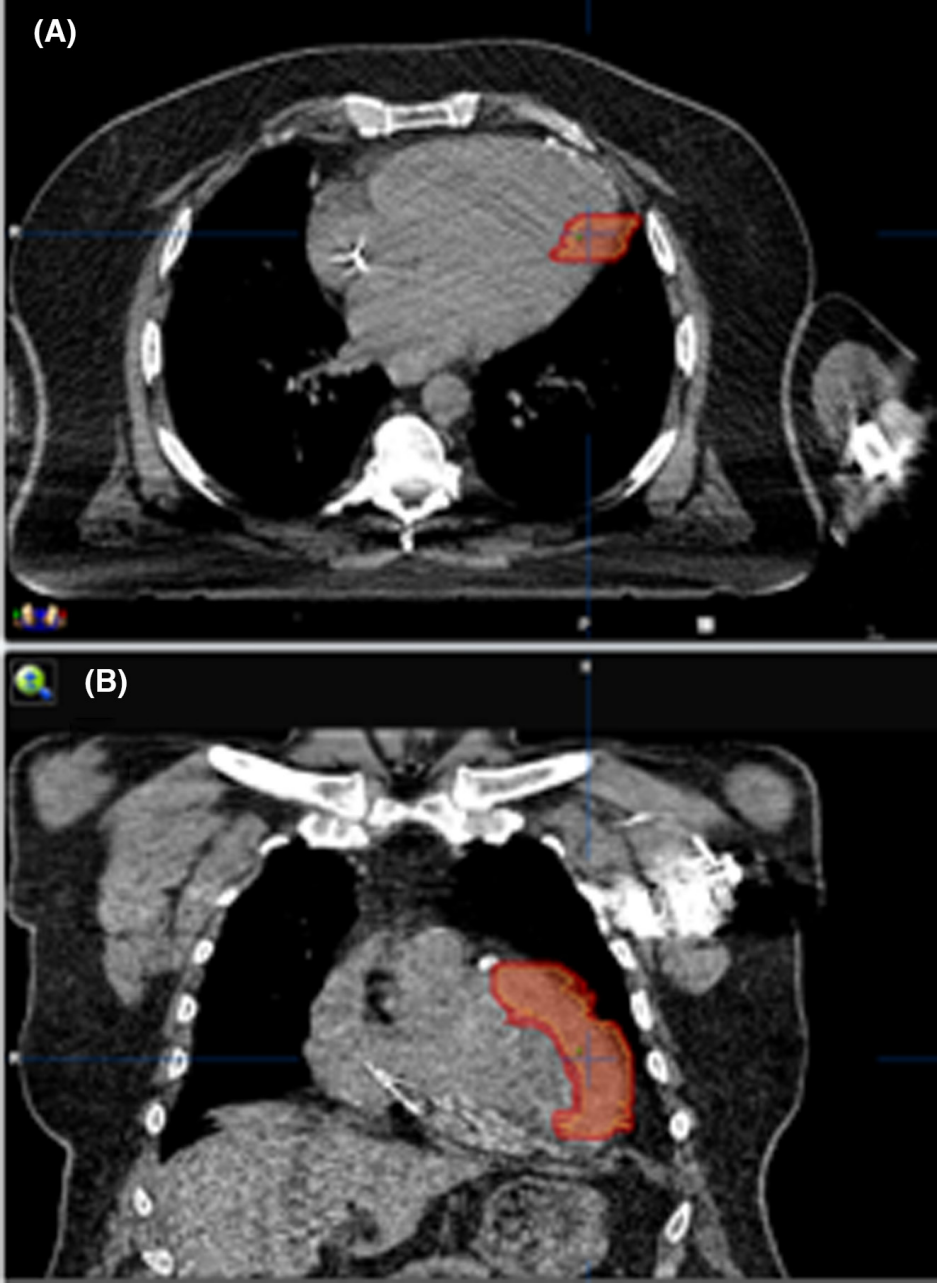
CT scan with the planning target volume highlighted in red. A: axial view. B: coronal view

We performed a series of CT scans with a free‐breathing CT and respiration‐correlated CT (4D CT). The 12‐lead ECG, 4D CT, and electroanatomic mapping were used to determine the planning target volume (PTV), which consisted of the gross target volume (GTV), the internal target volume (ITV) as well as 2 mm as an additional safety using the treatment planning system (TPS) iPlan RT® (BrainLAB) [Figure 3]. After the scar tissue was delineated (GTV), an additional area was added to account for the motion resulting from breathing as well as cardiac motion (ITV).

The PTV created using the TPS was targeted with a single dose of (25 Gy) high precision‐SBRT achieving maximal precision while sparing the surrounding organs at risk (OAT) such as lungs, esophagus, and surrounding myocardial tissue. SBRT was delivered using Novalis Tx® (Varian Medical Systems) using a dynamic conformal arc technique (6 MV‐SRS, 1000 MU/min). The patient was positioned on the linear accelerator using Exac Trac® (BrainLAB) X‐Ray 6D system as well as cone beam CT using a 6D verification threshold for translation of 0.5 mm and rotation of 0.5°.

SBRT was performed without complications or ICD dysfunction. During a one‐year follow‐up via ICD telemonitoring as well as outpatient clinic assessments, no need for adequate shocks, after a massive pre‐interventional VT burden. The LVEF improved to 31%. No complications related to SBRT were detected. The patient remained on Amiodarone, ß‐blockers, platelet inhibitor, diuretics, and a Sacubitril/valsartan combination.

## 3 CONCLUSION

Radiotherapy offers a feasible, safe, and effective therapy to VT storms and VF refractory to pharmacotherapy and ablation. We report a patient in whom radiotherapy was safely performed leading to complete elimination of ventricular arrhythmia during mid‐term follow‐up. Our results should be confirmed in a prospective multi‐center trial.

## AUTHOR CONTRIBUTIONS

**Alexander Wutzler:** Conceptualization; data curation; methodology; supervision; validation; writing – review and editing. **Borris Tiedke:** Data curation. **Mohamed Osman:** Conceptualization; supervision. **Reinhard Wurm:** Conceptualization; methodology.

## CONFLICT OF INTEREST

All authors declare no conflict of interest.

## CONSENT

Written informed consent was obtained from the patient to publish this report in accordance with the journal's patient consent policy.

## Data Availability

Data sharing is not applicable to this article as no new data were created or analyzed in this study.
